# The Healthy Eating Index and oral health among adults: a cross-sectional study from an Iranian RaNCD cohort data

**DOI:** 10.1038/s41598-023-37168-z

**Published:** 2023-07-20

**Authors:** Arash Mohammadi, Mitra Darbandi, Yahya Pasdar, Mahsa Mohebi, Negin Kamari, Narges Ziaei, Farid Najafi

**Affiliations:** 1 Research Center for Environmental Determinants of Health (RCEDH), Health Institute, Kermanshah University of Medical Sciences, Kermanshah, Iran; 2 Periodontology Department, Dental School, Kermanshah University of Medical Sciences, Kermanshah, Iran; 3 Kermanshah University of Medical Sciences, Kermanshah, Iran; 4 Cardiovascular Research Center, Kermanshah University of Medical Sciences, Kermanshah, Iran

**Keywords:** Diseases, Risk factors

## Abstract

This study was conducted to investigate the association between decayed, missing, and filled teeth (DMFT) index and nutritional status measured by Healthy Eating Index 2015 (HEI-2015), in Iranian adults. In this cross-sectional study, data from the Ravansar non-communicable diseases cohort study were analyzed. DMFT index was employed as a measurement of oral health. The HEI-2015 score was calculated based on data obtained from Food Frequency Questionnaire and categorized into quartiles. Linear regression models were used to assess the association between HEI-2015 and DMFT. From total of 7549 participants with the mean age of 45.65 ± 7.70, 3741 of them were female (49.56%). The mean of DMFT in the highest quartile of HEI-2015 was lower than the lowest quartile (12.64 ± 7.04 vs. 14.29 ± 7.54, *P* < 0.001). The mean of DMFT in subject who had higher socioeconomic status (SES (was significantly lower than those with low SES (*P* < 0.001). The mean of DMFT in the lowest quartile of HEI-2015 was significantly lower than in the highest quartile, after adjusting for confounding variables (ß = − 0.11, 95% CI − 0.54, − 0.30). The increasing dairy intake (β = − 0.08, 95% CI − 0.13, − 0.03) was associated with decreasing DMFT score and increasing refined grains (β = 0.20, 95% CI 0.02, 0.35) and sodium (β = 0.07, 95% CI 0.02, 0.12) intake was significantly associated with increasing DMFT score. A healthy diet was associated with a decrease in DMFT score in the studied population. Following a healthy diet is recommended for oral health.

## Introduction

The status of teeth and the digestive system have an essential role in the body's health status, and it requires regular evaluation. One of the most common index for assessing dental caries and dental treatment needs is DMFT. It has been used for about 75 years and counts the number of decayed, missing and filled teeth^1^. A meta-analysis study in 2018 indicated that the DMFT index of Iranian children and adults is 2.30 and 8.60, respectively^2^. Many studies have shown that there is an association between tooth loss and chronic diseases such as obesity, diabetes (T2DM), cardiovascular diseases (CVDs), some kinds of cancers, and all-cause mortality^3–6^.

Nutrition is a factor that has a significant correlation with DMFT^7,8^. The ability to chew is reduced in people who lose more teeth. Therefore, there may be changes in their dietary choices including reduced consumption of solid foods such as fruits and vegetables, nuts, and cooked meats. This can lead to the deficiency in essential nutrients^9–12^.

The Healthy Eating Index (HEI) is a valid index for the measurement of diet quality. It was developed by the US Department of Agriculture to monitor the intakes of the US population. The algorithm of HEI-2015 scoring is based on 2015–2020 Dietary Guidelines for Americans (DGA), using recommended intakes for food groups and nutrient, which are related to health^13^. The HEI-2015 index measures two crucial aspects of nutrition: First, adequacy by measuring nine foods items; and second, moderation for dietary intakes by measuring four food items^13,14^. Since there are few investigations about DMFT and nutritional status in Iran, this study was conducted to investigate the association between DMFT index and nutritional status based on HEI-2015 among Iranian adults.

## Methods

### Study design and participants

This cross-sectional study was conducted in 2021 using data from the baseline phase of the Ravansar non-communicable disease (RaNCD) cohort study in Ravansar, Kermanshah province, western Iran. The RaNCD study is part of a Prospective Epidemiological Research Studies in Iran (PERSIAN), which has been started by enrolling 10,047 adults aged 35–65 since 2014. Ravansar is a district with both urban and rural areas, located in Kermanshah province in the west of Iran and holds a population of about 50,000. The detailed methodology and design of the RaNCD study has been published in 2019^15^. Participants included all subjects from the baseline phase of the RaNCD study (n = 10,047). Participants with dentures (n = 2457) and missing data (n = 41) were excluded. Finally, 7549 subjects were examined.

### Data collection

Using a validated questionnaire, all required information was collected by well-trained personnel of the cohort center through face-to-face interviews. Demographic information, including age, sex, marital status, socio-economic status (SES), and smoking, was recorded online in an electronic data collection form. The standard Persian cohort questionnaire was used to assess the level of physical activity. This questionnaire has 22 questions about sports, work, and leisure—related activities on an average weekday and has been completed as a self-report.

### DMFT score measurements

The DMFT index was employed to measure oral health in this study. The DMFT score measured as the total number of permanent teeth that were decayed (D), missing (M), and filled (F).

### Healthy Eating Index 2015

Nutritional information extracted from the Food Frequency Questionnaire (FFQ) was applied to calculate the HEI-2015 scores^16^. The HEI- 2015 was calculated based on the method described by Krebs-Smith et al.^14^ HEI-2015, which encompasses 13 food items. Nine of these 13 items are emphasized to be consumed in adequate quantities which include whole fruits, total fruits, total protein foods, seafood and plant proteins, greens and beans, total vegetables, whole grains, dairy products, and fatty acids. Therefore, participants with the highest intake were given the highest point. The refined grains, sodium, added sugars and saturated fats should be consumed in moderation, and participants with the lowest intake were given the highest point. Accordingly, all participants received a score for each food item, a point from 0 to 10. Finally, the score of all items is added together and the final score is calculated as a number from 0 and 100 (Table 1).

**Table 1:** Healthy Eating Index—2015 (Intakes between the minimum and maximum standards are scored proportionately).

Component	Standard for maximum score	Standard for minimum score of zero	Maximum points
Adequacy			
Total fruits^a^	≥ 0.8 cup equivalent per 1000 kcal	No Fruit	5
Whole fruits^b^	≥ 0.4 cup equivalent per 1000 kcal	No whole fruit	5
Total vegetables	≥ 1.1 cup equivalent per 1000 kcal	No vegetables	5
Greens and beans	≥ 0.2 cup equivalent per 1000 kcal	No Dark–Green vegetables or legumes	5
Whole grains	≥ 1.5 cup equivalent per 1000 kcal	No whole grains	10
Dairy^c^	≥ 1.3 cup equivalent per 1000 kcal	No dairy	10
Total protein foods^d^	≥ 2.5 cup equivalent per 1000 kcal	No protein foods	5
Seafood and plant proteins^e^	≥ 0.8 cup equivalent per 1000 kcal	No seafood or plant proteins	5
Fatty acids^f^	(PUFAs + MUFAs)/SFAs ≥ 2.5	(PUFAs + MUFAs)/SFAs ≤ 1.2	10
Moderation			
Refined grains	≤ 1.8 oz equivalent per 1000 kcal	≥ 4.3 oz equivalent per 1000 kcal	10
Sodium	≤ 1.1 g per 1000 kcal	≥ 2.0 g per 1000 kcal	10
Added sugars	≤ 6.5% of energy	≥ 26% of energy	10
Saturated fats	≤ 8% of energy	≥ 16% of energy	10
Total score			100

^a^Includes 100% fruit juice.

^b^Includes all forms except juice.

^c^Includes all milk products, such as fluid milk, yogurt, and cheese, and fortified soy beverages.

^d^Includes legumes (beans and peas).

^e^Includes seafood, nuts, seeds, soy products (other than beverages), and legumes (beans and peas).

^f^Ratio of poly- and mono-unsaturated fatty acids (PUFAs and MUFAs) to saturated fatty acids (SFAs).

### Statistical analysis

Data were analyzed using Stata software, version 14.2 (Stata Corp, College Station, TX, USA). Baseline characteristics of participants across quartiles of the HEI-2015 and DMFT score were reported as mean ± standard deviation for continuous variables and as percentages for qualitative variables. To compare differences across HEI-2015 quartiles and DMFT, we used the one-way ANOVA and Chi square test. Linear regression models were applied to determine associations between HEI-2015 and DMFT score. All statistical analyzes were considered significant according to *P*-value of < 0.05 with 95% confidence intervals (CIs).

### Ethical approval and consent to participate

The Ethics Committee of Kermanshah University of Medical Sciences approved the design of this study (code: KUMS.REC.1399.067). Participants provided oral and written informed consent. Written informed consent was obtained from all subjects prior to enrollment in the study and all methods were carried out in accordance with relevant guidelines and regulations.

## Results

A total of 7549 participants, with a mean age of 45.65 ± 7.70 years, were enrolled. Compared with those in the lowest quartile, participants in the highest quartile of HEI-2015 were younger (*P* < 0.001). Overall, 3808 (50.44%) were male, 6851 (90.75%) were married, and 858 (11.47%) were current smokers. Participants with the highest HEI-2015 had fewer current smokers than the first quartile (Q1 = 40.44% vs. Q4 = 11.42%, *P* < 0.001). While the average DMFT was 13.33 ± 7.28, the score was lower among those with higher HEI-2015(*P* < 0.001) (Table 2).

**Table 2:** Baseline characteristics according to the Healthy Eating Index- 2015 quartiles.

Variables	Total (n = 7549)	HEI 2015 quartiles				*P* value
		Q1 (n = 2152)	Q2 (n = 1947)	Q3 (n = 1847)	Q4 (n = 1603)	
Age (year), mean ± SD	45.65 ± 7.70	46.65 ± 7.87	45.36 ± 7.53	45.29 ± 7.75	45.10 ± 7.46	< 0.001
Sex (%)						
Male	3808 (50.44)	1097 (28.81)	994 (26.10)	953 (25.03)	764 (20.06)	0.090
Female	3741 (49.56)	1055 (28.20)	953 (25.47)	894 (23.90)	839 (22.43)	
Marital status, n (%)						
Married	6851 (90.75)	1917 (27.98)	1771 (25.85)	1707 (24.92)	1456 (21.25)	< 0.001
Single	370 (4.90)	123 (33.24)	100 (27.03)	88 (23.78)	59 (15.95)	
Widowed/divorced	328 (4.34)	112 (34.15)	76 (23.17)	52 (15.85)	88 (26.83)	
Socio-economic status, n (%)						
1 (lowest)	2404 (31.86)	997 (41.47)	645 (26.83)	448 (18.64)	314 (13.06)	< 0.001
2	2573 (34.10)	697 (27.09)	670 (26.04)	657 (25.53)	549 (21.34)	
3 (Highest)	2569 (34.04)	458 (17.83)	629 (24.48)	742 (28.88)	740 (28.80)	
Physical activity (Met-h/day), n (%)						
Light	2225 (29.47)	534 (24.00)	607 (27.28)	565 (25.39)	519 (23.33)	< 0.001
Moderate	3557 (47.12)	976 (27.44)	894 (25.13)	884 (24.85)	803 (22.58)	
High	1767 (23.41)	642 (36.33)	446 (25.24)	398 (22.52)	281 (15.90)	
Smoking, n (%)						
Current	858 (11.47)	347 (40.44)	206 (24.01)	207 (24.03)	98 (11.42)	< 0.001
Former	577 (7.71)	184 (31.89)	156 (27.04)	128 (22.18)	109 (18.89)	
Never	6048 (80.82)	1605 (26.54)	1569 (25.94)	1495 (24.72)	1379 (22.80)	
Decayed teeth, mean ± SD	3.60 ± 4.16	3.60 ± 3.91	3.57 ± 4.11	3.65 ± 4.35	3.60 ± 4.31	0.014
Missed teeth, mean ± SD	8.19 ± 6.40	9.76 ± 7.13	8.10 ± 6.00	7.56 ± 5.99	6.90 ± 5.87	< 0.001
Filled teeth, mean ± SD	1.54 ± 2.65	0.92 ± 2.10	1.46 ± 2.53	1.82 ± 2.90	2.14 ± 2.95	< 0.001
DMFT, mean ± SD	13.33 ± 7.28	14.29 ± 7.54	13.12 ± 7.10	13.03 ± 7.27	12.64 ± 7.04	< 0.001
Number of teeth, mean ± SD	23.19 ± 6.26	21.82 ± 7.01	23.31 ± 5.85	23.77 ± 5.88	24.23 ± 5.80	< 0.001

*P- value was obtained one-way ANOVA and Chi square tests.

Table 3 presents the status of decayed, missing, filled teeth based on the baseline characteristics of the participants. The mean number of filled teeth was higher in women (*P* = 0.015). The mean number of decayed and missing teeth, as well as DMFT in participants with higher SES was significantly more than those with lower SES (*P* value for all < 0.001). The oral health measured by decayed, missing, filled, and DMFT was better in participants who flossed than in participants who did not (*P* value for all < 0.001). The mean of DMFT was 11.33 ± 5.95 in participants who brushed once or twice daily, and 17.05 ± 8.17 in participants who never brushed (*P* < 0.001).

**Table 3:** Baseline characteristics according to the condition of teeth.

Variables	Condition of the teeth, n (%)			
	Decayed	Missed	Filled	DMF
Age (year)				
35–50 years	3.46 ± 4.10	6.46 ± 5.03	1.86 ± 2.86	11.79 ± 6.50
51–65 years	3.99 ± 4.34	13.01 ± 7.32	0.63 ± 1.65	17.63 ± 7.64
*P* value	0.002	< 0.001	< 0.001	< 0.001
Sex				
Male	3.86 ± 4.50	8.43 ± 6.71	1.44 ± 2.56	13.73 ± 7.67
Female	3.34 ± 3.76	7.93 ± 6.10	1.64 ± 2.73	12.91 ± 6.84
*P* value	0.003	0.007	0.015	< 0.001
Marital status				
Married	2.51 ± 3.60	4.91 ± 4.17	2.16 ± 3.01	9.58 ± 5.83
Single	3.65 ± 4.19	8.27 ± 6.42	1.52 ± 2.63	13.46 ± 7.29
Widowed/divorced	3.72 ± 3.93	10.04 ± 6.92	1.15 ± 2.35	14.91 ± 7.30
* P* value	0.001	< 0.001	< 0.001	< 0.001
Socio-economic status				
1 (lowest)	4.283 ± 4.51	10.29 ± 7.11	0.62 ± 1.83	15.19 ± 7.81
2	3.85 ± 4.34	8.21 ± 6.10	1.18 ± 2.19	13.24 ± 7.18
3 (Highest)	2.70 ± 3.35	6.20 ± 5.32	2.76 ± 3.21	11.66 ± 6.37
*P* value	< 0.001	< 0.001	< 0.001	< 0.001
Physical activity (Met-h/day)				
Light	3.14 ± 3.74	7.73 ± 6.38	1.90 ± 2.87	12.77 ± 7.18
Moderate	3.45 ± 3.93	8.02 ± 6.26	1.66 ± 2.73	13.13 ± 7.02
High	4.49 ± 4.90	9.10 ± 6.63	0.84 ± 1.97	14.42 ± 7.79
*P* value	< 0.001	< 0.001	< 0.001	< 0.001
Smoking				
Current	5.17 ± 5.28	11.01 ± 7.82	0.73 ± 1.69	16.91 ± 8.41
Former	4.12 ± 4.38	10.43 ± 6.94	0.93 ± 1.95	15.49 ± 7.48
Never	3.32 ± 3.88	7.56 ± 5.95	1.71 ± 2.79	12.61 ± 6.88
*P* value	< 0.001	< 0.001	< 0.001	< 0.001
Flossing				
Yes	2.44 ± 3.37	4.95 ± 3.98	3.45 ± 3.40	10.84 ± 5.39
No	3.78 ± 4.24	8.69 ± 6.56	1.24 ± 2.38	13.71 ± 7.46
*P* value	< 0.001	< 0.001	< 0.001	< 0.001
Brushing				
Once or twice daily	2.86 ± 3.41	6.26 ± 4.85	2.19 ± 3.01	11.33 ± 5.95
Three times ≤ a day	2.43 ± 2.80	5.90 ± 4.81	2.38 ± 3.10	10.71 ± 5.30
Doesn’t brush	4.42 ± 4.83	12.11 ± 7.64	0.51 ± 1.58	17.05 ± 8.17
*P* value	< 0.001	< 0.001	< 0.001	< 0.001

*P- value was obtained one-way ANOVA and Chi square tests.

An inverse correlation was found between HEI-2015 and DMFT (r = − 0.10, *P* < 0.001). In addition, inverse correlation was found between HEI-2015 and missed teeth (r = − 0.172, *P* < 0.001) and filled teeth (r = − 0.170, *P* < 0.001).

The association between the HEI- 2015 and oral health assessed by linear regression model is shown in Table 4. Compared to quartile 1, the mean number of missing teeth in people in quartile 3 and 4 of HEI-2015 index was lower; (ß = − 2.21 (95% CI − 2.06, − 1.81) and ß = − 2.86 (95% CI − 3.27, − 2.45)), respectively. This association remained significant after adjusting for confounding variables including sex, age, SES, education level, and smoking. After adjusting for confounding variables, the mean of DMFT in the highest quartile of HEI-2015 was lower than in the lowest quartile (ß = − 0.11 (95% CI − 0.54, − 0.30)).

**Table 4:** Association between oral health and HEI- 2015.

Condition of the teeth	Quartiles of HEI 2015	Model I		Model II		Model III	
		β (95% CI)	*P* value	β (95% CI)	*P* value	β (95% CI)	*P* value
Decayed teeth	Quartile 1	Ref	–	Ref	–	Ref	–
	Quartile 2	− 0.03 (− 0.28, 0.23)	0.826	0.02 (− 0.23, 0.27)	0.896	0.32 (0.07, 0.57)	0.011
	Quartile 3	0.05 (− 0.20, 0.31)	0.687	0.10 (− 0.16, 0.35)	0.454	0.60 (0.34, 0.85)	< 0.001
	Quartile 4	0.01 (− 0.27,0.27)	0.993	0.11 (− 0.19, 0.34)	0.588	0.83 (0.56, 1.14)	< 0.001
Missed teeth	Quartile 1	Ref	–	Ref	–	Ref	–
	Quartile 2	− 1.67 (− 2.06, − 1.28)	< 0.001	− 1.12 (− 1.46,− 0.79)	< 0.001	− 0.63 (− 0.95,− 0.30)	< 0.001
	Quartile 3	− 2.21 (− 2.06, − 1.81)	< 0.001	− 1.64 (− 1.97, − 1.30 )	< 0.001	− 0.85 (− 1.18,− 0.51)	< 0.001
	Quartile 4	− 2.86 (− 3.27, − 2.45)	< 0.001	− 2.18 (− 2.53, − 1.83)	< 0.001	− 1.10 (− 1.42, − 0.72)	< 0.001
Filled teeth	Quartile 1	Ref	–	Ref	–	Ref	–
	Quartile 2	0.53 (0.37, 0.69)	< 0.001	0.43 (0.27, 0.59)	< 0.001	0.14 (− 0.05, 0.29)	0.059
	Quartile 3	0.89 (0.73, 1.05)	< 0.001	0.79 (0.63, 0.95)	< 0.001	0.31 (0.16, 0.47)	< 0.001
	Quartile 4	1.21 (1.04, 1.38)	< 0.001	1.10 (0.92, 1.25)	< 0.001	0.39 (0.24, 0.53)	< 0.001
DMFT	Quartile 1	Ref	–	Ref	–	Ref	–
	Quartile 2	− 1.16 (− 1.61, − 0.72)	< 0.001	− 0.68 (− 1.10, − 0.27)	0.001	0.05 (− 0.34, 0.44)	0.405
	Quartile 3	− 1.26 (− 1.71, − 0.81)	< 0.001	− 0.74 (− 1.16, − 0.33)	< 0.001	0.08 (− 0.03, 0.77)	0.749
	Quartile 4	− 1.64 (− 2.11, − 1.17)	< 0.001	− 1.01 (− 1.44, − 0.58)	< 0.001	− 0.11 (− 0.54, − 0.30)	0.047

Model I: Unadjusted; Model II: Adjusted for sex and age; Model III: Adjusted for sex, age, SES, flossing, brushing and smoking.

Association between the HEI-2015 components and DMFT by linear regression model is presented in Table 5. After adjusting for confounding variables, increasing dairy intake was associated with decreasing DMFT score. In addition, increasing refined grains and sodium intake was associated with increasing DMFT score.

**Table 5:** Association between DMFT and HEI- 2015 components.

Components of HEI	Crude model		Adjusted model*	
	β (CI 95%)	*P* value	β (CI 95%)	*P* value
Total fruits	− 0.06 (0.18, 0.06)	0.352	0.41 (0.31, 0.52)	< 0.001
Whole fruits	− 0.21 (− 0.35, 0.08)	0.002	0.33 (0.21, 0.45)	< 0.001
Total vegetables	− 0.12 (− 0.26, 0.03)	0.117	0.04 (− 0.09, 0.18)	0.889
Greens and beans	0.16 (0.04, 0.29)	0.010	0.24 (0.13, 0.35)	0.001
Whole grains	0.001 (− 0.10, 0.10)	0.980	0.04 (− 0.09, 0.17)	0.555
Dairy	− 0.12 (− 0.18, − 0.06)	< 0.001	− 0.08 (− 0.13, − 0.03)	0.006
Total protein foods	0.06 (− 0.08, 0.20)	0.401	0.28 (0.15, 0.41)	0.001
Seafood and plant proteins	− 0.18 (− 0.42, 0.06)	0.142	− 0.07 (− 0.30, 0.12)	0.526
Fatty acids	− 0.11 (− 0.16, − 0.05)	< 0.001	− 0.01 (− 0.06, 0.03)	0.621
Refined grains	0.29 (0.10, 0.47)	0.002	0.20 (0.02, 0.35)	0.016
Sodium	− 0.11 (− 0.17,00.05)	< 0.001	0.07 (0.02, 0.12)	0.009
Added sugars	− 0.14 (− 0.20, − 0.10)	< 0.001	− 0.34 (− 0.43, − 0.26)	< 0.001
Saturated fats	0.01 (− 0.05, 0.07)	0.753	− 0.02 (− 0.06, 0.04)	0.529

*Adjusted for sex, age, SES, flossing, brushing and smoking.

## Discussion

To the best of our knowledge, this is the first study that presents the oral health status, and examines the association between the Healthy Eating Index and oral health in a large sample of Kurdish adults. The major finding of the present study was that a healthy diet was significantly associated with a decrease in DMFT score. According to the results of this study, the mean of DMFT among people in the highest quartile of the HEI-2015 score was lower than in the lowest quartile (ß =− 0.11 (95% CI − 0.54, − 0.30)). In addition, after adjusting for confounding variables, increasing dairy and decreasing refined grains and sodium intake were associated with a decrease in DMFT score.

A study in American adults has shown, greater compliance with the DGA is associated with lower odds of untreated caries. The average coronal DMFT decreased as HEI-2015 scores increased, but trends were not consistent in different ethnic or racial groups. American adults who followed the HEI-2015 recommendations were less likely to have untreated coronal caries than those who did not follow the recommendations^17^.

Bawadi et al. have reported that poor diet was significantly associated with an increased risk of periodontal disease in Jordanian adults^10^. A cross-sectional study conducted by Al-Zahrani et al. on 12,110 individuals showed that individuals who maintained a healthy diet were less likely to have periodontitis compared with people who did not^18^. In our sample population, despite being provided with information about healthy nutrition in all district health centers, most of the people do not follow the guidelines of a healthy diet. Thus, the consumption of fruits and vegetables is less than the recommended and the consumption of salt, sugar and fats is higher than the allowed limit. It is noteworthy that the increase in food prices in recent years in Iran, may contribute to the lower consumption of some of the food groups such as fresh fruits and vegetables, nuts and proteins.

According to the findings of the present study, after adjusting for confounding variables, increasing refined grains and sodium intake was significantly associated with increasing DMFT score. Moreover, increasing dairy intake was significantly associated with decreasing DMFT score. Studies in Denmark and India have shown that dental plaque is lower in people who receive dairy products as recommended^19,20^. A prospective study in American adults also investigated the effect of dietary pattern on dental caries and found that a diet based on consuming more sugar and less dairy products increased the risk of dental caries^21^.

In the present study, it was found that mean DMFT score was higher in older people, men, participants with lower SES, and smokers. This difference in age groups may be due to mechanical changes in tooth decay due to aging, including changes in calcium absorption and cariogenic microbiota^21,26^. Similar to these findings, the study of Najafi et al.^22^ has shown the effect of socioeconomic inequality in dental caries in 17 provinces of Iran. A systematic review and meta-analysis study (2019) reported an increased risk of dental caries with increased tobacco smoking^23^. In addition, SES and current smoking were also related to HEI-2015. In this study, it was observed that people with higher SES are in higher quartiles of HEI-2015. However, the role of SES in people’s food choices and purchasing power is undeniable, and other studies have proven this association^24,25^. Therefore, these factors were adjusted as confounding variables in examining the association between HEI-2015 and DMFT. The association we found between oral health and HEI was independent of these confounding factors.

The results of this study showed that there is a need for more training in the region to increase compliance to a healthy diet. Regular dental examinations and trainings related to oral health also need to be strengthened in Kurdish population.

### Study strengths and limitations

One of the limitations of this study was its cross-sectional nature and therefore, causal associations cannot be established based on these findings. We were not able to measure or modulate the effect of genetic factors. A large sample size is one of the strengths of this study. We were able to control most potentially confounding variables.

## Conclusion

The finding of the present study showed that a healthy diet was significantly associated with a decrease in DMFT score. According to the results of this study, the mean of DMFT among people in the highest quartile of the HEI-2015 score was lower than in the lowest quartile.

In addition, after adjusting for confounding variables, increasing dairy intake was significantly associated with decreasing DMFT score and increasing refined grains and sodium intake was significantly associated with increasing DMFT score.

## Data Availability

The datasets used and/or analyzed during the current study are available from the corresponding author on reasonable request.
